# Spontaneous Crystallization in Athermal Polymer Packings

**DOI:** 10.3390/ijms14010332

**Published:** 2012-12-24

**Authors:** Nikos Ch. Karayiannis, Katerina Foteinopoulou, Manuel Laso

**Affiliations:** Institute of Optoelectronics and Microsystems (ISOM) and ETSII, Polytechnic University of Madrid (UPM), José Gutiérrez Abascal 2, 28006 Madrid, Spain; E-Mails: nkarayiannis@etsii.upm.es (N.Ch.K.); kfoteinopoulou@etsii.upm.es (K.F.)

**Keywords:** polymer, crystallization, entropy, phase transition, Voronoi tesselation, simulation, Monte Carlo, hard sphere, random packing, crystal morphology

## Abstract

We review recent results from extensive simulations of the crystallization of athermal polymer packings. It is shown that above a certain packing density, and for sufficiently long simulations, all random assemblies of freely-jointed chains of tangent hard spheres of uniform size show a spontaneous transition into a crystalline phase. These polymer crystals adopt predominantly random hexagonal close packed morphologies. An analysis of the local environment around monomers based on the shape and size of the Voronoi polyhedra clearly shows that Voronoi cells become more spherical and more symmetric as the system transits to the ordered state. The change in the local environment leads to an increase in the monomer translational contribution to the entropy of the system, which acts as the driving force for the phase transition. A comparison of the crystallization of hard-sphere polymers and monomers highlights similarities and differences resulting from the constraints imposed by chain connectivity.

## 1. Introduction

Crystallization and phase transitions in general play a key role in many processes related, among others, to material engineering, physics, chemistry and biology. Advances in crystallography, mainly through X-ray diffraction measurements, have provided significant information on crystal structures. However, how such crystals nucleate and grow and how processing history further affects the corresponding ordered morphologies remain open topics of intense scientific debate. While experimental, theoretical and modeling advances constantly enrich our fundamental understanding of the phenomenon in a wide range of physical systems [1–7], a plethora of key aspects remain unknown, especially with respect to the microscopic origins of crystallization. Computer simulations can greatly aid in this direction through systematic studies on ideal atomic and molecular systems under controlled conditions, which remain unattainable in conventional approaches. Such an “*in silico*” modeling approach, subject to obvious advantages and disadvantages compared to experiments, has been shown to be an invaluable research tool in the analysis of the highly complex process of crystallization.

Deep in the heart of numerical simulations lays the molecular model, which determines the level of detail and the corresponding approximations with respect to the way atoms and molecules are represented. Atomistic models incorporate highly detailed force fields to describe interactions between atoms, while coarse-grained ones sacrifice detailed information in favor of computational efficiency. In one of the simplest possible representations, atoms, either as monomeric entities or as part of molecular species, are treated as non-overlapping hard spheres. The hard-sphere model is obviously void of any kind of chemical information. However, because of its simplicity it stands as an invaluable simulation tool: It is accessible to analytical approaches, requires minimal computational resources and time, and can thus be employed under conditions which remain inaccessible to more detailed molecular models. Furthermore, the hard-sphere model allows us to discriminate and accurately identify the different governing factors (for example density or entropy) that affect various phenomena and physical processes. Ideally the knowledge gleaned from such simplified models could shed light onto the fundamental role of analogous mechanisms in much more complex physical and biological applications. Thus, it is not surprising that during the last decades an ever-growing body of simulations has successfully employed the hard-sphere model in studies of systems that range from colloids, microgels and granular materials to synthetic and biological polymers.

The study of how objects of different shapes and sizes arrange in a multidimensional space and of the corresponding packing morphologies has been in the spotlight of research since early historical times. During the last decades pioneering scientific contributions have been achieved in general packing with modeling studies having greatly benefited by the continuous advances in computer hardware and software.

Almost four centuries ago, Kepler conjectured that in three dimensional space the densest hard-sphere packing is that of a face centered cubic (fcc) lattice, a long-standing geometrical problem that has been addressed only recently in a series of papers by Hales and coworkers [8–10]. Equally interesting and perhaps more complex in its mathematical and physical formulation is the analogous problem of random packing: What is the maximum achievable density in the absence of order? Which are the salient characteristics of this state that could serve as a fingerprint for its identification? Under which conditions does an assembly of spheres transit between the amorphous and ordered phases? Many key aspects of random close (densest) packing of spheres were revealed by the pioneering experimental studies of Bernal and collaborators [11–14] while a rigorous definition of the maximally random jammed (MRJ) state has been provided more recently [15–18]. Over the years significant advances have provided a wealth of information about the state of jamming in a wide range of model physical systems [19–35].

Regarding phase transition in athermal packings, Onsager was the first to predict an anisotropic-nematic transition in hard rods as a result of the increase in entropy caused by ordering [36]. Crystallization in hard-sphere systems was initially reported by the independent simulations of Alder and Wainwright [37] and Wood and Jacobson [38]. Frenkel and collaborators studied and analyzed in detail the entropic mechanism behind phase transition in colloidal systems and model athermal packings at various conditions [39–51]. For packings of monomeric hard spheres of uniform size the calculated phase diagram identifies the freezing and melting points at ϕ_F_ = 0.494 and ϕ_M_ = 0.545, respectively [52]. It is now well established that above the melting point and given sufficient time an initially random packing of monodisperse hard spheres transits to the crystal phase [53–56]. Furthermore, crystallization is strongly affected by, among other factors, size polydispersity [39,57–61], microgravity [62,63], shear stress [64,65] and the presence of interfaces [66–69]. With respect to the morphology of the crystal phase, free energy calculations have shown that the face centered cubic (fcc) is thermodynamically more stable than the hexagonal close packed (hcp) lattice [45,70], albeit by a small margin [71]. However, in accordance with Ostwald’s rule of stages [72], the formation of a random hexagonal close packing (rhcp) is regularly observed in experiments on colloidal systems [63,73–75] and in simulations of monomeric hard spheres [55,56,76] since such stacking is configurationally closer to the random arrangement than the pure fcc and hcp lattices. The presence of defects in the crystal boundaries, the very small differences in the free energy of the competing crystal structures and the very slow dynamics at such high densities hinder the formation of a perfect crystallite. From the modeling perspective these kinetic hindrances are aggravated by computational limitations in system size and simulation time [56].

Random hard-sphere packings are further characterized by short-range order in the form of polytetrahedral structures with fivefold symmetry [35,56,77–79]. In crystal phases such structural morphologies are strongly correlated with multiply twinned planes at crystallite boundaries [55,56,76]. The evolution of fivefold local symmetry during crystal nucleation and growth in dense packings of monomeric hard spheres has been studied extensively through event-driven Molecular Dynamics (edMD) [56] and Monte Carlo (MC) simulations [1]. Based on these modeling results, a microscopic interpretation of hard-sphere crystallization proposes a competition between short-range order and crystallization in dense packings, where the formation of long-lived fivefold structures could frustrate the growth of crystallites [1,55,56].

Recent advances in experimental and simulation techniques have contributed to the detailed analysis and characterization of the phase behavior and self-assembly in packings of objects with highly complex shapes [32,80–86]. Among these systems lie the athermal polymer packings, consisting of chains of hard-sphere monomers. Macromolecules are characterized by a wide spectrum of characteristic time and length scales, which render their study very challenging from the modeling perspective, especially in atomistic detail. Furthermore, holonomic constraints applied to constituent monomers by chain connectivity, and inter-chain topological constraints in the form of entanglements, render the dynamical, conformational, mechanical and rheological properties of polymers distinctly different than the ones of monomeric analogs. A pertinent question with respect to dense packings of athermal polymers is how chain connectivity affects the maximally random jammed (MRJ) state and the disorder–order transition (crystallization) of hard spheres at high volume fractions. During the last years a growing body of research work has focused on such open topics related to the intrinsic features of athermal polymer packing and to the self-assembled morphologies of associated systems [33,87–97].

In the present manuscript we review our latest results from extensive Monte Carlo (MC) simulations on the crystallization in dense packings of freely-jointed chains of tangent hard spheres of uniform size [98–100]. In particular, we describe in detail the modeling methodology for the creation of athermal polymer configurations and the metrics adopted to characterize local order. Particular emphasis is placed on identifying the entropic origins of the phase transition and on comparing with the corresponding trends in monomeric analogs [55,56]. The paper is organized as follows: Section 2 describes the systems studied, the MC algorithm and the novel descriptors introduced to quantify local environment of each site. Section 3 presents the main results on the disorder-order phase transition, the self-assembly of crystallites and the corresponding structural differences with respect to the random phase. The manuscript concludes with Section 4, where the key findings are summarized with a description of potential applications on the self-assembly of crystals for more complex systems.

## 2. Methodology

### 2.1. System Studied/Monte Carlo Algorithm

Athermal polymer packings consist of freely jointed, linear chains of tangent hard spheres. Monomers are treated as non-overlapping spheres of diameter σ. Tangency implies that the bond length *l* is equal to the sphere diameter. This condition is numerically imposed within a tolerance in bond length. Comparisons carried out by allowing the bond length to fluctuate in the interval *l* ∈ [σ, σ + 10^−4^] [91] and in the interval *l* ∈ [σ, σ + 10^−8^] showed no difference in the crystal growth and nucleation as well as in the self-assembly of the ordered morphologies. The freely-jointed model allows for full flexibility in the conformations as there are no constraints in bond bending and torsion (dihedral) angles. However, it has been shown that, due to strong excluded-volume interactions, bond bending angles and torsion angles tend to adopt specific geometric arrangements, which become increasingly more favorable as packing density increases [88,89,91–93]. Such a conformational tendency in bonded geometry leads to major changes in the long-range characteristics of chains: Their size shrinks significantly once the marginal scaling regime is reached [89,93,94]. As a consequence, in the vicinity of the MRJ state (concentrated regime), polymer dimensions become so collapsed that a significant fraction of chains form closed loops (cyclic long-range conformations) [88].

The employed Monte Carlo scheme consisted of the following mix of moves: (i) reptation (10%); (ii) end-mer rotation (10%); (iii) configurational bias (20%); (iv) inter-chain reptation (25%); (v) internal libration (34.98%); (vi) simplified end-bridging (sEB, 0.1%) and (vii) simplified intramolecular end-bridging (sIEB, 0.1%), where the percentages in parenthesis denote the attempt probabilities of each move. All local moves (i–v) are executed in a configurational bias pattern [101–103], according to which multiple trial positions, whose number increases with volume fraction, are attempted for each displaced site. This algorithm significantly increases the average computational time per MC step, but, in contrast to the conventional MC, it guarantees short-range equilibration of chains even at packing densities well above the melting point [91]. Long-range equilibration is achieved by the pair of chain-connectivity altering moves sEB and sIEB [91], which are based on the original end-bridging (EB) move [104] for atomistic polymer systems. Based on the tangency condition, sEB and sIEB proceed by deleting and forming bonds between properly selected pairs of spheres instead of displacing trimers [91,105]. Through this rapid re-arrangement long-range equilibration is achieved within modest computational time even in the close vicinity of the MRJ state; in fact the acceptance rate and accordingly the performance of the chain-connectivity altering moves increase with concentration [90,91]. In addition, the sEB and sIEB moves allow for polydispersity in chain lengths to be considered, which is controlled by casting the simulations in the *n*_at_*n*_ch_*VT*μ ensemble, where *n*_at_ is the total number of spheres, *n*_ch_ is the number of chains, *V* is the volume of the simulation cell, *T* is temperature and μ is the spectrum of relative chemical potentials of all chain species except two which are singled out as reference species [91,104]. In our simulations two different chain length distributions were implemented: A uniform one in the closed interval [*N*_av_ (1 − Δ), *N*_av_ (1 + Δ)], where *N*_av_ is the average chain length and Δ is the reduced half width of the distribution divided by *N*_av_, and a most probable (Flory) one with the shortest allowed chain length set at *N*_min_.

All simulations were executed in cubic cells with periodic boundary conditions applied in all dimensions. Three different polymer systems were modeled, each one containing a total of 1200 hard spheres: (i) *N*_av_ = 12, Δ = 0.5; (ii) *N*_av_ = 12, *N*_min_ = 3 and (iii) *N*_av_ = 24, Δ = 0.5. Additional simulations conducted with simulation cells of 3000 sites to investigate the effect of system size on crystallization and on the formation of ordered morphologies showed no appreciable qualitative and quantitative differences. Initial configurations were generated at very low volume fractions using fully equilibrated, atomistic polyethylene structures [106–110] as templates by performing short equilibration steps to ensure the absence of overlaps between hard spheres. These dilute cells, filled with overlap-free athermal polymer chains, were then used as initial structures in MC simulations with isotropic shrinkages of the cell dimensions being attempted at frequent intervals until a target volume fraction was reached. Such cell volume reductions were accompanied by an affine repositioning of chains based on the relative position of their end with respect to the box origin and the amplitude of the attempted box shrinkage. Configurations of the *N*_av_ = 12 system were further generated at selected packing densities by splitting all chains of a *N*_av_ = 24 configuration in half to guarantee that the structural characteristics of the initial random packings and the phase transition were not affected by the generation protocol of the modeling procedure. In production simulations system snapshots and ensemble statistics were recorded every 2 × 10^5^ MC steps, while the total simulation time exceeded 1 × 10^10^ steps at the higher densities. Due to very long runs required to observe crystallization at volume fractions in the vicinity of the MRJ state, modeling studies were necessarily limited to packing densities of ϕ = 0.56, 0.58, 0.60 and 0.61 above the melting point. More details on the algorithm and the procedure to generate and equilibrate random packings of athermal polymer chains can be found in [92].

For comparison purposes parallel sets of simulations for analogous monomeric systems were carried out by event-driven Molecular Dynamics (edMD). The edMD algorithm used was a minor modification of the conventional edMD technique, which proceeds on a simple collision-by-collision basis until a preset number of collisions is reached [111]. Initial configurations of monomeric hard spheres were generated by deleting all bonds from random chain packings and by subsequently performing an edMD equilibration. Because the observation of crystallization in monomers requires much shorter simulations than in chain systems, a larger set of eight statistically uncorrelated MD trajectories of monomeric samples was produced at each packing density.

### 2.2. Analysis of Local Structure, Voronoi Cell and Characteristic Crystallographic Element Norm

Once a large number of system configurations (frames) is collected, the analysis proceeds by a detailed characterization of the local environment around each site. An accurate and highly discriminating descriptor is required to quantitatively describe the degree of randomness as well as the appearance and propagation of ordered nuclei corresponding to specific crystal structures. Existing descriptors of local structure include the widely used pair radial distribution function, *g*(*r*) [111], and a set of rotationally invariant measures, which are defined as combinations of spherical harmonics [112]. *g*(*r*) provides detailed information on the radial characteristics of the atomic or particulate system under study while rotationally invariant measures detect orientational deviations with respect to perfect local order.

Recently, we qualitatively and quantitatively analyzed the local structure of athermal packings through a novel scheme that consists of two main steps: (i) Identification of the local environment around each sphere through a Voronoi tessellation and by measuring the shape and size of the corresponding Voronoi cell, and (ii) application of a novel structural descriptor based on the concept of the characteristic crystallographic element (CCE), as used in crystallography [113,114].

In structural characterization via Voronoi tessellation the set of neighbors closer to a reference site than to any other sites is identified. This task was performed with the *qhull* algorithm [115,116], which yields full information about the vertices, edges and faces of the Voronoi polyhedron around every site. Once the tessellation is completed, the corresponding Voronoi cells are constructed. In the simplest approach the local density around each hard sphere is calculated as the inverse of the volume of the corresponding Voronoi polyhedron [117]. A more detailed topological analysis can be performed with respect to the shape and size of each Voronoi cell through the calculation of the mass moment of inertia tensor **I** with all vertices being treated as equivalent point unit masses

(1) $$I=\frac{1}{n_{\text{ver}}}\sum_{i=1}^{n_{v\text{er}}}\left({r_{\text{i}}}^{2}\delta -r_{\text{i}}r_{\text{i}}\right)$$

with *n*_ver_ being the number of vertices of the polyhedron, **r***_i_* is the position vector of vertex *i* with respect to the center of mass of the polyhedron and **δ** is the unit second order tensor. Equation 1 is written in dimensionless form. The mass moment of inertia tensor provides a quantitative description of the shape of a rigid body and of the spatial distribution of its mass [89,118–120]. The internal, co-moving principal axis system of the Voronoi polyhedron is defined by the normalized eigenvectors (**e**_1_, **e**_2,_**e**_3_). Once the Voronoi tessellation is completed, the internal principal axes system is determined for each Voronoi cell from the coordinates of its vertices. The three real eigenvalues of the intertia tensor *I*_1_, *I*_2_ and *I*_3_ (*I*_1_ ≥ *I*_2_ ≥ *I*_3_) correspond to the principal moments of inertia. The inertia tensor provides useful information on the shape and size of the Voronoi cell and accordingly on the local environment around each hard sphere. Based on the eigenvalues, a coarse-grained ellipsoid can be constructed with the lengths of the semiaxes being calculated as:

(2) $$L_{1}=\sqrt{\frac{5}{2}\left(I_{2}+I_{3}-I_{1}\right)}$$

with semiaxis lengths *L*_2_ and *L*_3_ being calculated in an analogous fashion as in Equation 2 under cyclic permutation of the indices. As global shape measures of the coarse-grained ellipsoid the following were computed:

asphericity:
  (3) $$b=\frac{1}{2}\left(I_{1}+I_{2}\right)-I_{3}$$
acylindricity:
  (4) $$c=I_{1}-I_{2}$$
and relative shape anisotropy:
  (5) $$k^{2}=4\left(1-3\frac{I_{2}I_{3}+I_{3}I_{1}+I_{1}I_{2}}{{\left(I_{1}+I_{2}+I_{3}\right)}^{2}}\right)$$

These measures are defined so that the lower the values of *b*, *c* and *k*^2^ the closer the resemblance to spherical, cylindrical and isotropic shapes, respectively.

Once a system configuration was recorded in the course of MC or edMD simulations, a Voronoi tessellation was performed to identify the characteristics of the corresponding polyhedra including a shape analysis based on asphericity, acylindicity and relative shape anisotropy. These global shape measures for each individual Voronoi cell can be directly compared with the analogous measures for the trapezo-rhombic dodecahedron and the rhombic dodecahedron, which are the characteristic Voronoi polyhedra arising from tessellation of hcp and fcc lattices, respectively. Thus, a systematic analysis of the shape measures of the Voronoi cells at each instance provide a reliable estimate of the current state of the athermal packing as well as of possible phase transition (crystallization). In addition, a change in the local environment around each sphere, quantified by the Voronoi global shape measures, can be directly related to changes in translational entropy, quantified in turn by sphere mobility.

The second descriptor of local structure, the characteristic crystallographic element (CCE) norm for a given configuration of point-like atoms around a reference atom *j,* defined by the corresponding position vectors, quantifies both the orientational and radial similarity of this set of sites with respect to a specific ordered structure. This reference crystal structure is characterized by a unique, and thus distinguishing, set of crystallographic elements each of which, in turn, consists of a set of distinct elements of the corresponding point symmetry group. The complete mathematical formulation of the CCE norm of site *j* with respect to a reference crystal structure *X*, denoted as ɛ*_j_^X^* can be found in [89,98,100]. Algorithmically, the method proceeds by identifying the set of orientation axes in the internal coordinate system that minimizes the value of ɛ*_j_^X^*. For an ordered site *j* of perfect X crystal structure ɛ*_j_^X^* = 0, any deviation will lead to CCE values greater than zero. By construction and due to the highly discriminating nature of the CCE norm, a site with high similarity to a given ordered structure *X* (ɛ*_j_^X^* → 0) will necessarily possess a high norm value with respect to any alternative *Y* crystal structure (ɛ*_j_^Y^* ≫ 0).

Once the minimum CCE norm is calculated for each site in system, an order parameter with respect to perfect order *X* can be calculated as

(6) $$S^{X}=\int_{0}^{{ɛ}^{\text{thres}}}P\left({ɛ}^{X}\right)\text{d}{ɛ}^{X}$$

where *P*(ɛ*^X^*) is the probability distribution function of CCE norm ɛ*^X^* and ɛ^thres^ is a threshold value below which a site is considered to possess *X*-like order. Trial tests suggest that a value ɛ^thres^ = 0.245 of is adequately small to discriminate between different crystal types but also large enough to correctly identify the disorder-order transition in initially random packings and the emergence of specific crystal morphologies.

The hcp and fcc crystals are the two competing structures that arise when dense hard-sphere packings crystallize. Thus, the CCE norms (ɛ^hcp^, ɛ^fcc^ ) and the corresponding order parameters (*S*^hcp^, *S*^fcc^) for each were calculated with respect to these ordered structures. As the CCE-based analysis is highly discriminating between different crystal lattices, the degree of ordering τ^c^ can be estimated as the total number of sites with either hcp or fcc structural similarity (τ^c^ = *S*^hcp^ + *S*^fcc^). Additional measurements were conducted to detect sites with fivefold local symmetry, a structural motif which is favored at high packing densities and constitutes an alternative local arrangement to hcp and fcc crystals [55,56]. We should note that while the CCE-based descriptor is used here to compare with the hcp, fcc and fivefold symmetries, by incorporating the proper distinguishing set of crystallographic elements and operations, it can be used to identify any emerging crystal structure. As in the case of the Voronoi shape measure characterization, application of the CCE norm allows for an accurate description of the local environment around each site and for a precise identification of a potential disorder-order phase transition at high volume fractions.

## 3. Results and Discussion

### 3.1. Phase Transition of Athermal Systems: Effect of Chain Connectivity

**Figure 1:**
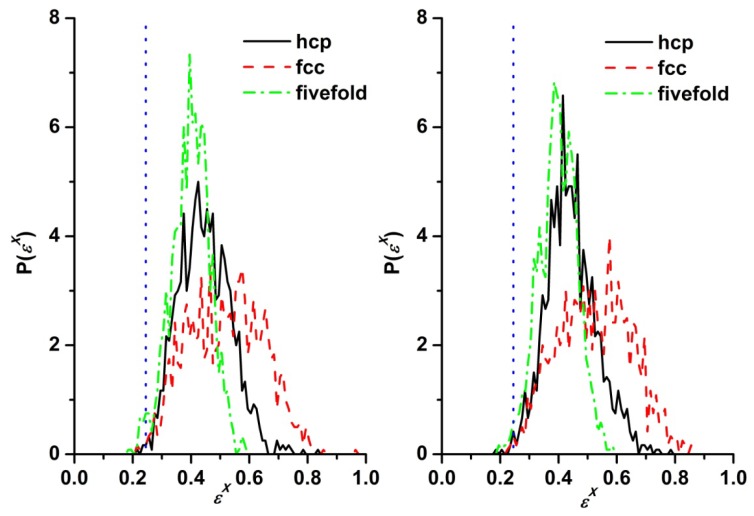
Characteristic crystallographic element (CCE)-based distribution with respect to hexagonal close packing (hcp), face center cubic (fcc) and fivefold symmetries as obtained from Monte Carlo (MC) simulations on the *N* = 12 system at ϕ = 0.56: (**left panel**) at the beginning and (**right panel**) at the end of the simulation. Also shown with vertical dotted lines are the threshold values of the CCE analysis (ɛ^thres^ = 0.245).

**Figure 2:**
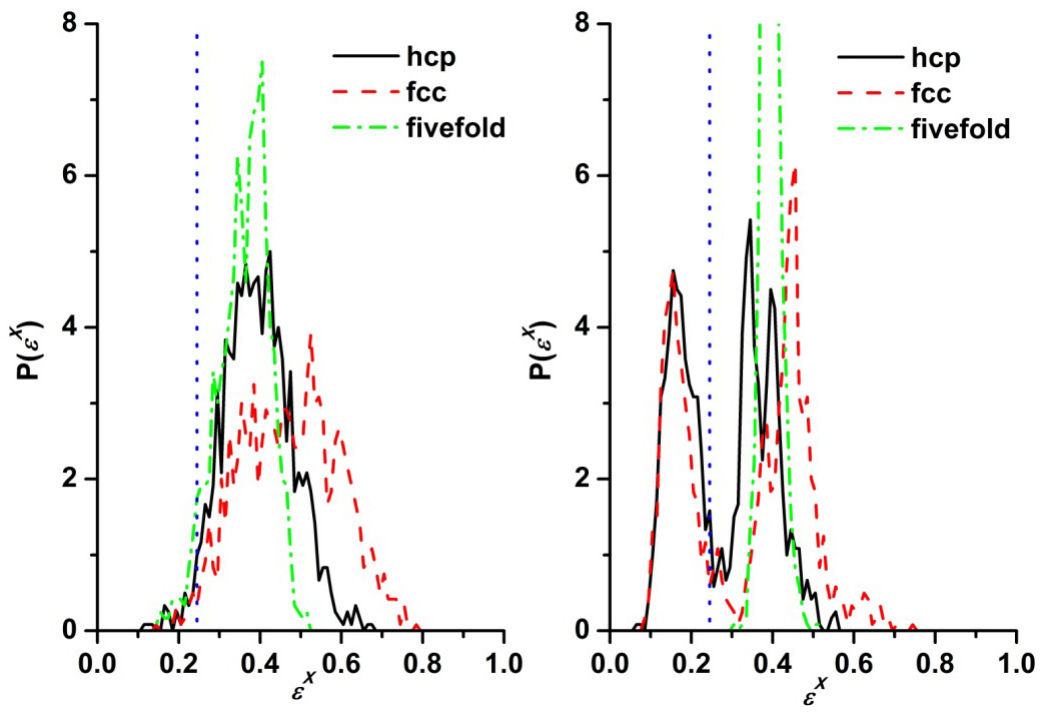
Same as in Figure 1 but at a packing density of ϕ = 0.61.

The analysis of local structure based on the concepts of the Voronoi cells and of the CCE-based norm was performed at equally spaced frames of the long MC (or edMD) trajectories over all computer-generated samples and at all packing densities. Figures 1 and 2 present the CCE norm distribution of sites for the *N* = 12 chain system at ϕ = 0.56 and 0.61, respectively. The CCE norm was calculated with respect to the hcp, fcc and fivefold symmetries and is presented here for two different frames, one very close to the beginning (left panel) and one at the end (right panel) of the MC simulation. The vertical dotted lines denote the CCE-based threshold (ɛ^thres^ = 0.245) below which a site is assigned to one of the reference structures (hcp, fcc or fivefold). According to Equation 2, the fraction of sites with specific *X* local order corresponds to the part of the CCE-based distribution (*P*(ɛ*^X^*)) that lies below the threshold value.

According to the data reported in Figure 1, at a volume fraction of ϕ = 0.56 there exist no appreciable differences in norm distributions for all three different reference structures (hcp, fcc and fivefold). Furthermore, the fraction of sites with highly ordered local structure, in other words the fraction of sites with hcp- or fcc-norms below the threshold value, is very small and does not change throughout the MC simulation. Thus, it can be safely concluded that the athermal chain packing (*N* = 12) shows no signs of phase transition and remains in the original amorphous (random) state. The situation is quite different at the higher density (ϕ = 0.61). While the shapes of the initial CCE distributions are similar to those at ϕ = 0.56 for all three structures, the same is not true at later stages. A very clear shift of the hcp and fcc CCE distributions to much lower values is evident, with many sites possessing CCE norms well below the critical threshold. In parallel, the shape of the corresponding CCE distributions is significantly altered. With respect to the fivefold local structure, the distribution becomes narrower and the average shifts to higher values, which implies that in the final state for the *N* = 12 polymer system there exist no sites with fivefold local symmetry. In parallel, the distributions of the hcp and fcc norms adopt a bidisperse shape with peaks at low and high values stemming from the discriminating nature of the CCE norm: By construction, an hcp-like site (low hcp CCE norm) is characterized by a high fcc CCE norm and *vice versa* [56,98,100]. For this specific sample (*N* = 12, ϕ = 0.61), the fraction of sites with hcp- and fcc-like local structures in the final state are very similar. By calculating the corresponding fractions it can be concluded that crystallization occurs for the athermal polymer packing at ϕ = 0.61.

**Figure 3:**
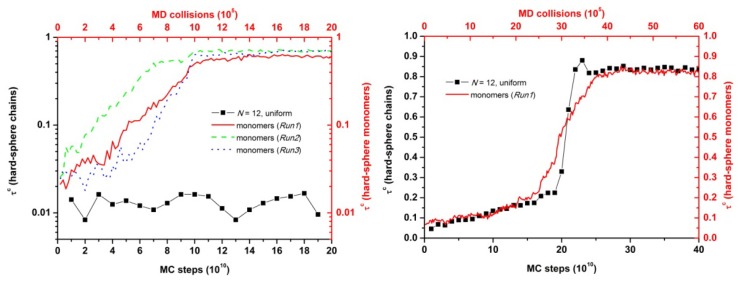
Crystallinity, τ^c^, as a function of MC steps and Molecular Dynamics (MD) collisions as obtained from simulations of freely-jointed chains of tangent hard spheres. (*N*_av_ = 12, uniform chain length distribution) and on monomeric hard spheres, respectively through application of the CCE norm, at (**left**) a packing density of ϕ = 0.56 where *Run1*, *Run2* and *Run3* denote MD trajectories starting from Frames 0, 10^10^ and 19 × 10^10^, respectively, of the corresponding MC trajectory by deleting all existing bonds, (**right**) ϕ = 0.61, where in *Run1* monomeric MD simulation is initiated from Frame 10^9^ of the corresponding MC trajectory.

Information obtained from the CCE-norm distribution also allows the calculation of crystallinity (degree of ordering),τ^c^, as a function of steps (or number of collisions) from MC and edMD simulations on athermal chains and on hard-sphere monomers, respectively. Panels (a) and (b) in Figure 3 present the evolution of crystallinity as a function of MC steps (number of collisions for edMD). We should note that while one could transform both measures (steps and collisions) into a common reference framework of CPU time, such mapping would only provide technical information about the computational cost of each method. The application of stochastic, non-physical (but highly efficient) MC algorithms for polymer chains prevents the extraction of any kind of dynamical information related to chain motion and to the kinetics of phase transition. While crystallinity results are presented here for the *N* = 12 system with uniform distribution of lengths the phase behavior of athermal polymer chains remains the same also for the other two systems (*N* = 24 with uniform distribution and *N* = 12 with Flory distribution of chain lengths). As seen in Figure 3 (left panel) at the volume fraction of ϕ = 0.56, which lies just above the melting point (ϕ_M_ = 0.545), hard sphere monomers of uniform size show a clear disorder-order transition while the corresponding athermal chains remain in the original amorphous state throughout the simulation. Evidently, at this packing density, chain connectivity suppresses crystallization. However, as concentration increases, polymer packings spontaneously evolve into a stable crystal phase. This trend is clearly shown in Figure 3 (right panel): Initially the degree of crystallinity, as quantified by the CCE norm, remains low (τ^c^ = 0.05) for both chains and monomers as expected for random (amorphous) packings. However, as MC (MD) simulations evolve, a sharp ordering transition occurs as crystallinity adopts high values (τ^c^ = 0.83), which are very similar for chain and monomeric packings. In the final stable crystal phase, the majority of sites adopt a highly ordered structure of either hcp or fcc character.

**Figure 4:**
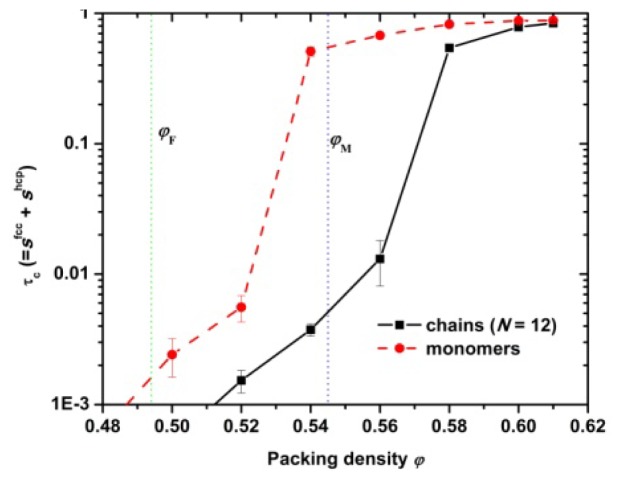
Crystallinity (degree of ordering), τ^c^, as a function of packing density, ϕ, for freely-jointed chains of tangent hard spheres (*N* = 12) and for hard sphere monomers. Vertical green and blue dotted lines indicate the freezing (ϕ_F_) and melting points (ϕ_M_), respectively, for hard sphere monomers of uniform size.

Figure 4 shows the dependence of crystallinity on packing density for hard-sphere chains and monomers. At a range of volume fraction near and above the melting point hard sphere monomers of uniform size crystallize while the corresponding polymer systems remain amorphous. As packing density increases, the difference in crystallinity between chains and monomers progressively gets smaller. At the highest studied concentration (ϕ = 0.61) CCE-based crystallinity is, within statistical error, the same between athermal chains and monomers. Thus, it can be safely concluded that at high volume fractions chain connectivity has no appreciable effect on the ability of hard spheres to crystallize given adequate simulation time. However, according to the present simulations near the melting point, connectivity frustrates phase transition of chain packings, which, in sharp contrast to monomeric analogs, remain predominantly amorphous.

### 3.2. Crystal Morphologies in Ordered Packings of Athermal Chains

**Figure 5:**
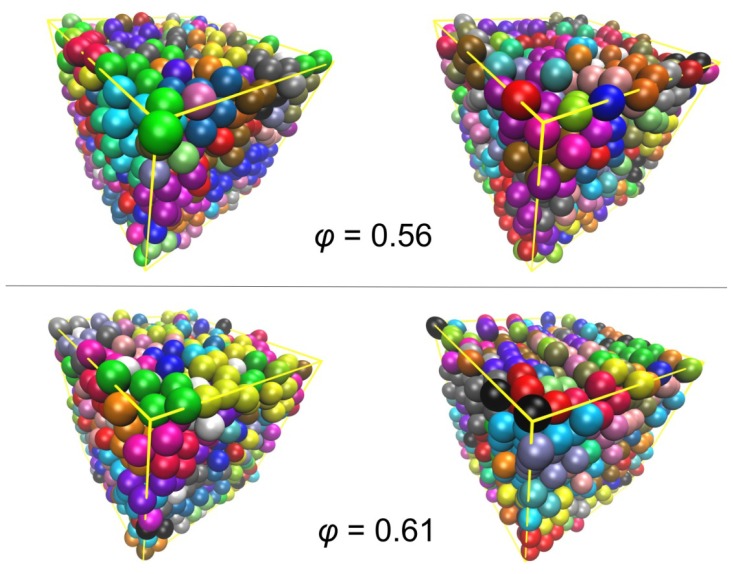
System configurations of the hard-sphere chain packing (*N* = 12) as obtained from MC simulations at packing densities of ϕ = 0.56 (upper panels) and ϕ = 0.61 (lower panels). Snapshots at the beginning and at the end of the simulation appear in the left and right panels, respectively. Spheres are color-coded according to the parent chain molecule. Image created with the VMD software [121].

From the simulation results presented in Section 3.1 it is evident that once a critical volume fraction is reached, which lies at higher concentrations compared to monomers, athermal packings of freely-jointed chains of hard spheres transit from the initial amorphous (random) to the final crystal (ordered) phase. In the present section, we study in detail the structural features of the characteristic ordered morphologies that arise during athermal polymer crystallization. Figure 5 shows some representative snapshots as obtained for the *N* = 12 system with uniform distribution of chain lengths at ϕ = 0.56 (upper panel) and 0.61 (lower panel) at the start (left side) and at the end (right side) of the MC simulations. In the system snapshots of Figure 5, spheres are colored according to the index of the chain to which they belong. While no significant ordering is observed in the structures at ϕ = 0.56, a visual inspection at ϕ = 0.61 confirms the ordering of the spheres and the formation of layers, as is clearly visible in the final configuration at ϕ = 0.61.

**Figure 6:**
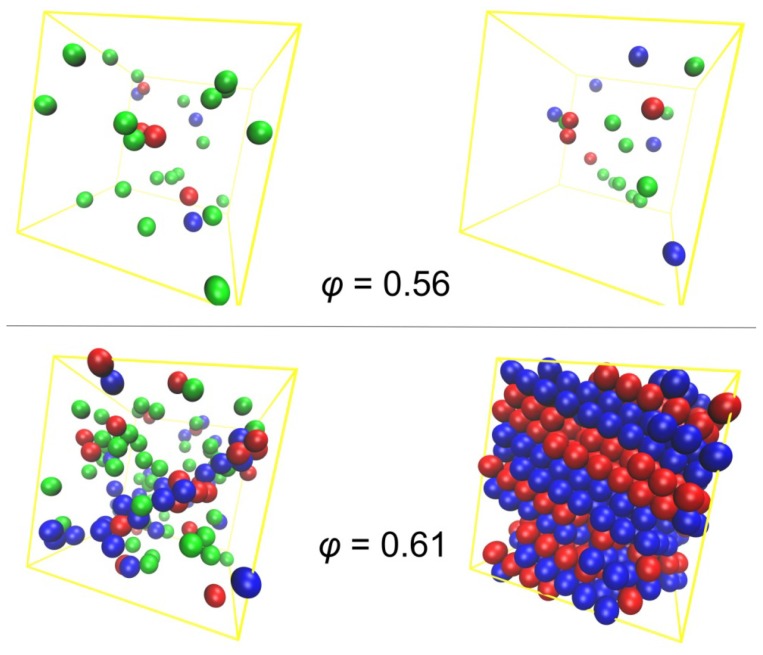
Same as in Figure 5, but following a different visualization pattern. Sites are color-coded according to the following scheme: Spheres with hexagonal close packing (hcp), face center cubic (fcc) and fivefold similarities are shown in blue, red and green colors, respectively; for visualization purposes all remaining spheres are omitted. Image created using the VMD software [121].

The loss of positional and radial randomness and the formation of well-ordered morphologies during crystallization are depicted more vividly once we adopt a visualization scheme based on the information obtained by the CCE analysis. In Figure 6, spheres are color-coded according to the corresponding CCE-norm for hcp, fcc and fivefold symmetries for the same chain configurations as in Figure 5. At ϕ = 0.56 no particular change is observed in the population of the ordered sites (hcp or fcc symmetry), which remains at low levels: At ϕ = 0.56 packings of freely-jointed chains of tangent hard spheres remain amorphous. At ϕ = 0.61, as expected, the initial fraction of sites with ordered local environment in the random phase is significantly higher than at ϕ = 0.56, and the same conclusion can be drawn regarding the sites with fivefold symmetry. The latter finding is in full agreement with corresponding results on random packings of monomeric hard spheres above the freezing transition, where the fraction of fivefold sites increases linearly with packing density [55,56]. In the final stable crystal phase at ϕ = 0.61, alternating layers of almost exclusive hcp- or fcc- type are formed. These stack-faulted ordered morphologies possess a unique stacking direction and are further accompanied by an absence of fivefold sites.

**Figure 7:**
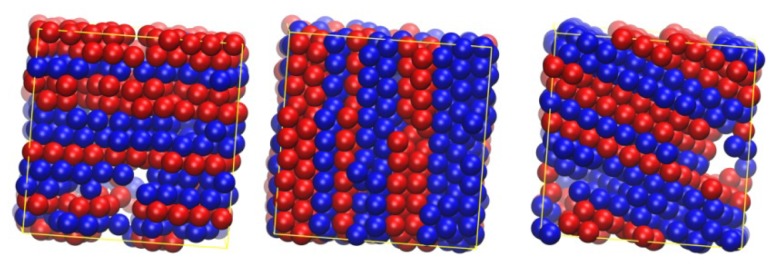
System configurations corresponding to the final crystal morphologies as obtained from MC simulations on *N* = 12 athermal polymer packing where chain lengths follow the Flory (most probable) distribution at volume fractions: (left) ϕ = 0.58, (middle) ϕ = 0.60 and (right) ϕ = 0.61. Spheres with high hcp and fcc similarities, according to the CCE norm, are depicted in blue and red colors, respectively. Image created using the VMD software [121].

Analogous snapshots of the final crystal phases as obtained from MC simulations on the *N* = 12 polymer system, where chain lengths obey the Flory (most probable) distribution, are shown in Figure 7 at packing densities of ϕ = 0.58, 0.60 and 0.61. As in the case of the polymer packing characterized by the same average chain length but by a uniform chain-length distribution, stable crystal structures correspond to layered morphologies of alternating hcp and fcc character with a unique stacking direction. Furthermore, sites with fivefold local symmetry are absent in the ordered state.

According to the results presented here, crystal morphologies of dense assemblies of athermal polymers correspond to randomly stacked hexagonal close packings (rhcp), and no ordered structures were found of exclusive fcc (or hcp) character. As mentioned in the introduction, this trend is in accordance with the Ostwald rule of stages [72], as the rhcp morphology is structurally and thermodynamically closer to the random phase than the pure fcc crystal. Accordingly, in a second step, a transition is expected from the (metastable) rhcp to the (stable) fcc phase. However, in all present MC simulations on athermal polymer packings, no such transition occurs even if the total simulation time at cases exceeds by orders of magnitude the time required for crystallization (disorder-order transition), which is not unexpected in view of the tiny entropic difference between rhcp and fcc structures. Similar conclusions have been drawn for corresponding packings of monodisperse hard-sphere monomers as the rhcp structure is shown to be the prevailing morphology [55,56]. In parallel, crystallization processes of athermal packings consisting of chains and monomers show appreciable differences. For chains, crystal morphologies are free of defects such as twinning which appear in ordered phases of monomeric hard spheres. Furthermore, while even for monomers the rchp is the prevailing crystal structure in the majority of samples, ordered morphologies still exist, which are characterized by a clear prevalence of fcc (or hcp) sites. Studies are in progress to investigate in a systematic fashion the effect of chain connectivity on the established crystal morphologies and on the short-range order in the form of fivefold local symmetry.

### 3.3. Evolution of the Voronoi Cell during Hard-Sphere Crystallization

**Figure 8:**
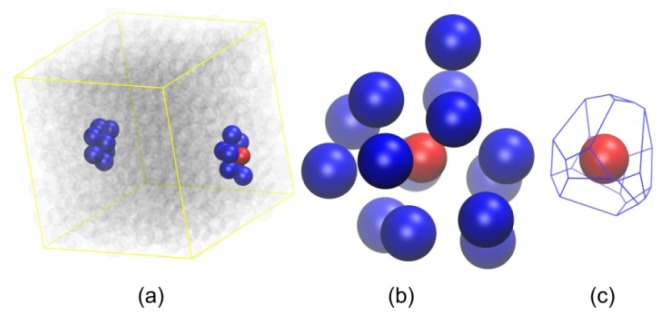
Illustration of the procedure adopted for the construction of the Voronoi polyhedron around a reference site. In the specific example a sphere site is randomly selected from a system configuration obtained from MC simulations on *N* = 12 chain system at ϕ = 0.56. (**a**) Reference sphere and corresponding nearest neighbors, as calculated from Voronoi tessellation, are shown in red and blue colors, respectively; sphere coordinates are subjected to periodic boundary conditions. All remaining spheres are transparent for visualization purposes. (**b**) Reference sphere and nearest Voronoi neighbors with coordinates fully unwrapped in space and sphere radii reduced for clarity. (**c**) Voronoi polyhedron enclosing the reference sphere. Image created with the VMD software [121].

In the introduction we proposed a second method to identify crystallization by analyzing the changes in the local environment through a systematic study of the shape and size of the Voronoi cell around each sphere site. This approach is presented through a series of illustrations starting in Figure 8 with the parent hard-sphere configuration where all nearest neighbors of a reference site are identified through the Voronoi tessellation (Figure 8a,b). The enclosing Voronoi cell of the reference site is shown in Figure 8c. Once the Voronoi cell is constructed, the moment of inertia tensor **I** is calculated according to Equation 2. In the next step the Voronoi cell is mapped to a coarse-grained ellipsoid with semiaxes given by Equation 3. Finally, global shape measures of the simplified representation can be readily calculated through Equations 4–6.

**Figure 9:**
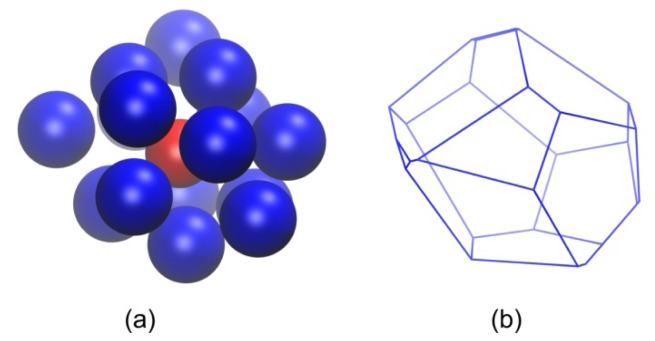
System visualizations showing: (**a**) a reference hard sphere and the nearest Voronoi neighbors; (**b**) the corresponding Voronoi polyhedron. The local environment of the reference sphere is predominantly amorphous (ɛ^hcp^ = 0.6730, ɛ^fcc^ = 0.7207). Global shape measures for the Voronoi cell: asphericity *b* = 2.52, acylindricity *c* = 1.42 and relative shape anisotropy *k*^2^ = 0.0472.

**Figure 10:**
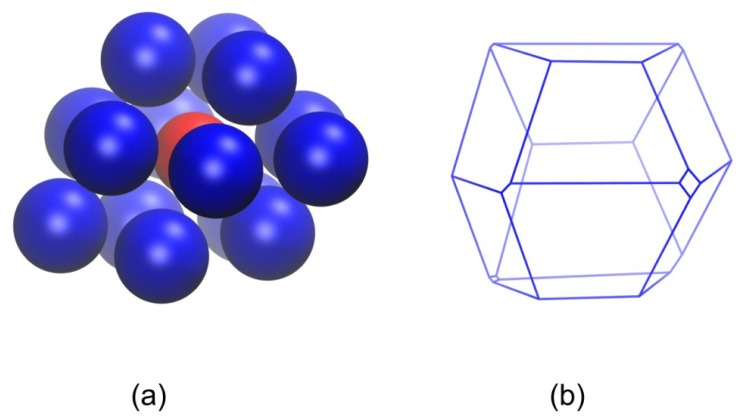
Same as in Figure 9 but for a reference hard sphere with well-ordered hcp-like local structure (ɛ^hcp^ = 0.0675). Global shape measures for the Voronoi cell: asphericity *b* = 1.34, acylindricity *c* = 0.262 and relative shape anisotropy *k*^2^ = 0.0146.

**Figure 11:**
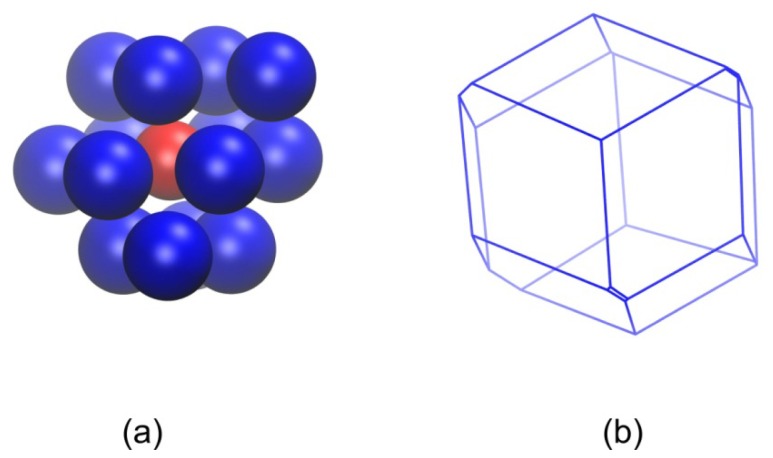
Same as in Figure 9 but for a reference hard sphere with well-ordered fcc-like local structure (ɛ^fcc^ = 0.0845). Global shape measures for the Voronoi cell: asphericity *b* = 0.620, acylindricity *c* = 0.261 and relative shape anisotropy *k*^2^ = 0.00508.

An estimate of the local density around each sphere site can be obtained as the reciprocal of the volume of the enclosing Voronoi polyhedron. Since the volume of the simulation cell remains constant during the simulation and the Voronoi tessellation is a space-filling geometrical procedure, the average local density of the system does not change during the whole simulation time and consequently during the phase transition. However, significant qualitative and quantitative changes occur in the shape of the Voronoi cells as the chain assembly crystallizes spontaneously and ordered morphologies are formed. Figures 9, 10 and 11 show the parent sphere configuration along with the corresponding Voronoi cell for sites, which possess amorphous and well-ordered hcp-like and fcc-like local structures, respectively.

**Figure 12:**
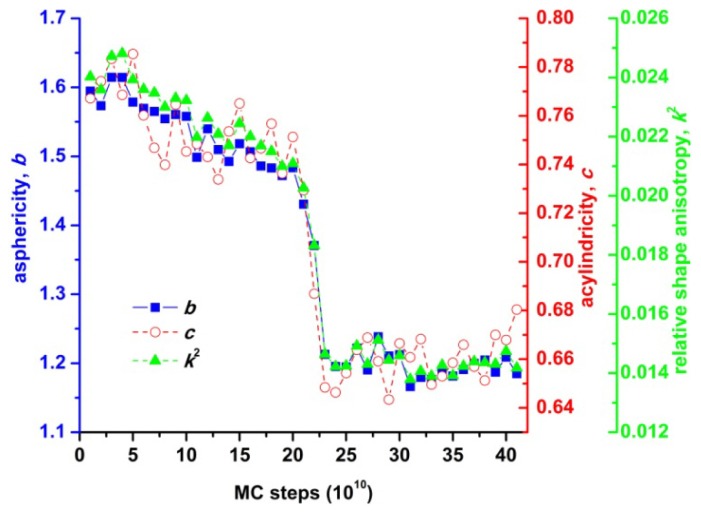
Average asphericity, *b*, acylindricity, *c*, and relative shape anisotropy, *k*^2^, as a function of MC steps from simulations of freely-jointed chains of tangent hard spheres (*N* = 12 with uniform distribution of chain lengths) at ϕ = 0.61. Global shape measures are averaged over all 1200 coarse-grained ellipsoids, each one having semi-axes as obtained from the eigenvalues of the moment of inertia tensor of the corresponding Voronoi polyhedron.

Visual comparison of the shapes of the Voronoi cells depicted in Figures 9, 10 and 11 clearly shows that the local environment around each site undergoes significant changes as the packing evolves from the amorphous to the crystal phase. The Voronoi polyhedra corresponding to well-ordered crystal structures have more symmetrical and more spherical shapes. Thus, while there is no appreciable difference in the average local density, as quantified by the Voronoi volume, between the amorphous and crystal phases, the shape of the local environment around each site is profoundly altered during the phase transition [98,99]. Such structural changes during the phase transition can be made quantitative by plotting the global shape measures (asphericity, acylindricity and relative shape anisotropy) as a function of MC steps (Figure 12).

All shape measures of deviation from isotropy, averaged over all Voronoi cells for each system configuration, decrease monotonically as the MC simulation advances. According to the data shown in Figure 3, for the *N* = 12 system (uniform chain length distribution) a sharp phase transition (crystallization) occurs at around 20 × 10^10^ MC steps. It is exactly the same regime where the values of asphericity, acylindricity and relative shape anisotropy of the Voronoi polyhedra show a precipitous decline. In the final stable crystal phase all values of shape measures are significantly lower than the initial ones of the random phase. Based on the above, it can be safely concluded that during crystallization of athermal polymer packings, on average, the local environment around each sphere site becomes more symmetric and more spherical. Thus, a detailed geometrical analysis of the Voronoi polyhedra can shed light on the structural changes that occur during the phase transition. Such a methodological approach could be complementary to more refined structural descriptors like the CCE-based norm. In addition, such shape transformations of the local environment around each sphere site can be directly connected with local dynamics and consequently with translational entropy.

The present analysis based on the Voronoi polyhedra can further serve as the basis for a descriptor, which would potentially identify shape similarities with respect to specific Voronoi cells of reference crystal structures.

### 3.4. Entropic Origins of Crystallization in Hard-Sphere Chain Packings

**Figure 13:**
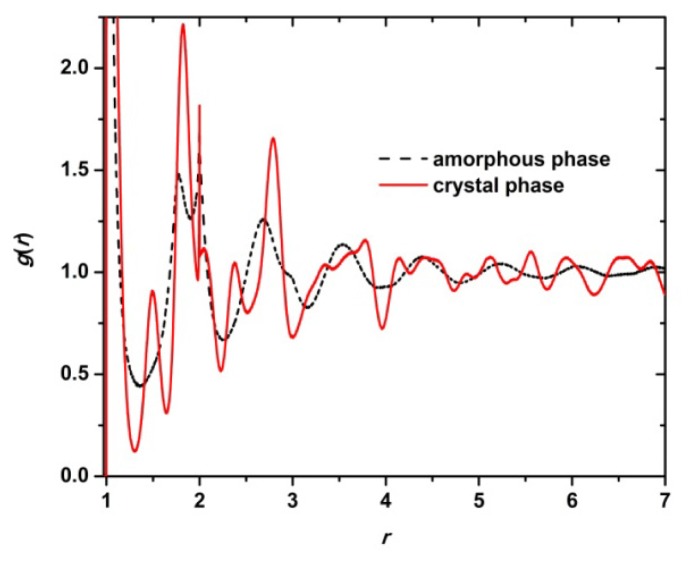
Total pair radial distribution function, *g(r)*, as a function of radial distance, *r*, in the initial amorphous and the final crystal phases as obtained from MC simulations on the *N* = 12 chain system with uniform length distribution at ϕ = 0.61.

In isolated athermal systems, a phase transition can only be driven by an increase in entropy. Accordingly, in athermal packings of chain molecules, entropy is the driving force for crystal nucleation and growth. The conformational contribution entropy is actually reduced as a result of sphere arrangements adopting specific conformations both in bonded and non-bonded terms. This trend is easily identified by comparing the pair radial distribution function, *g(r)*, in the initial random and final ordered phases as seen in Figure 13.

Characteristic peaks appear in the ordered phase, especially near contact. At the same time, long-range correlations features in the pair distribution unambiguously point towards the emergence of the ordered phase. Even if a more refined measure of pair correlation would be required to capture the anisotropic features of layered crystals, *g*(*r*) points to a clear loss of conformational entropy during crystallization. Similar conclusions can be drawn for the orientational contribution of entire chains to entropy. While the population of oriented chains is quite limited, so that the change in the average orientation vector is very small, orientational entropy is nevertheless reduced in polymer crystals compared to random chain packings [98].

**Figure 14:**
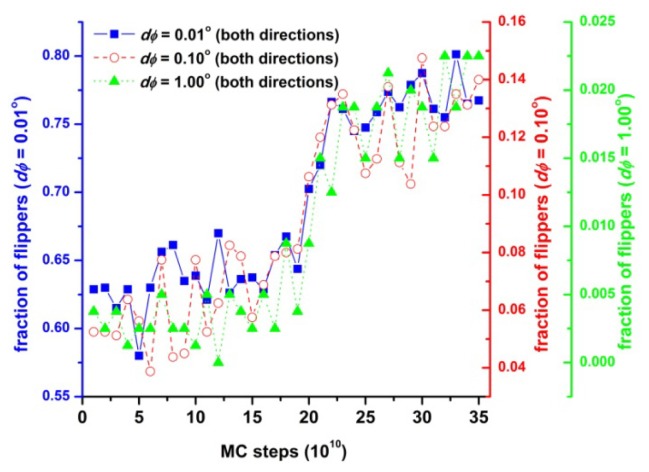
Fraction of sites (flippers), which can perform a flip-like move clockwise and counter-clockwise of amplitudes dφ = 0.01, 0.10 and 1.00° as a function of MC steps from simulations on the *N* = 12 chain system with uniform chain length distribution at ϕ = 0.61. Reproduced from [98] with permission from The Royal Society of Chemistry.

The two previous sources of entropy loss must be more than compensated for by an independent mechanism of entropy gain. In Section 3.3 we have reported that the local environment around each hard sphere becomes more isotropic as the crystal phase appears. In order to establish a connection between shape transformation of local structure and entropy increase, we first studied local sphere dynamics. In this direction, and given that MC simulation provide no dynamical information, we resort to the concept of “flipper” originally employed to identify the jamming transition in polymer packings [92,94]. “Flipper” is a term used to denote a sphere, which can perform a flip-like move, which obeys the holonomic chain constraints and does not lead to overlaps with any other sphere of the system. Here, we employ the concept of flipper to study how the ability of hard spheres to move locally is affected by the shape transformations of the local environment, which take place during crystallization. Figure 14 shows the fraction of sites (flippers), which can perform a flip-like move of specific amplitude, dφ in both directions (clockwise and counter-clockwise) as a function of MC steps corresponding to the same simulation trajectory of Figures 3 and 12. As simulation progresses, the population of sites which can move freely in their local vicinity increases. This trend is especially apparent around 20 × 10^10^ MC steps, a regime which marks the disorder-order transition. Thus, as the local environment becomes more spherical and more symmetric monomers are able to explore more efficiently the free volume that surrounds them. Consequently, the translational entropy of the athermal polymer system increases during crystallization. This increase in translational entropy is large enough to compensate for the losses in conformational and orientational entropy, and is thus responsible for crystallization.

**Figure 15:**
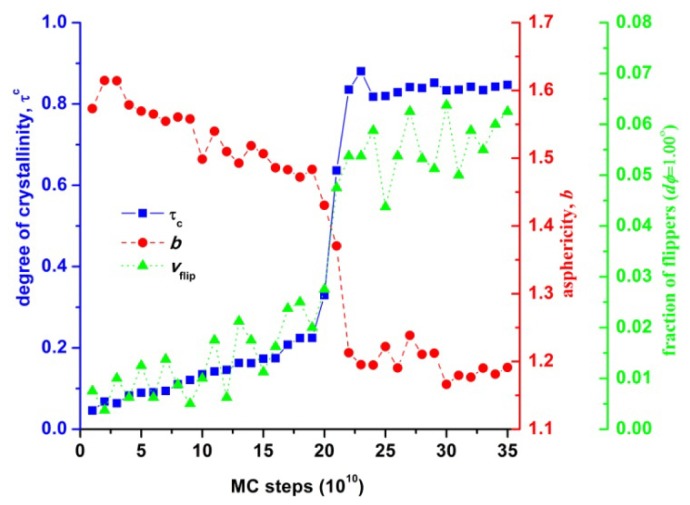
Asphericity, *b*, averaged over all Voronoi polyhedra, degree of crystallinity, τ^c^, and fraction of flippers (dφ = 1.00°) as a function of MC steps from simulations on the *N* = 12 chain system with uniform chain length distribution at ϕ = 0.61.

In order to better understand the strong correlation between the shape transformation of the local environment and the increase in translational entropy during crystallization, Figure 15 summarizes the evolution of asphericity (averaged over all Voronoi polyhedra), fraction of flippers (of amplitude dφ = 1.00°) and crystallinity as a function of MC steps. The data shown confirm that during phase transition (indicated by the sharp increase of crystallinity), as the local environment becomes more spherical (indicated by the sharp decline of asphericity), translational entropy increases (indicated by the sharp increase of the flipper population). The large increase in translational entropy is thus the driving force for the athermal polymer crystallization as in the case of monomeric analogs.

## 4. Conclusions

We have reviewed recent studies on the phase transition and self-assembly of crystal morphologies from extensive simulations of packings of freely-jointed chains of tangent hard spheres of uniform size. The key finding is that once a critical packing density is reached, athermal polymer chains crystallize, just as monomers do, in spite of the additional constraint set by chain connectivity. However, at volume fractions very close to the melting point, chain connectivity does indeed frustrate crystallization; for the systems studied at ϕ = 0.61, polymer packings remain amorphous while monomeric analogs show a clear phase transition. The exact origins of the frustration along with the extent of the effect of connectivity on crystallization are under investigation.

Above a critical packing density, which is higher than for monomeric systems, and given sufficient simulation time, polymer configurations self-assemble into well characterized ordered morphologies of predominately rhcp character. Such crystals consist of stack-faulted alternating layers of hcp or fcc type with a single stacking direction and never show twinning. For all chain systems studied so far no transition to a pure fcc (or hcp) crystal was observed during the allowed simulation time. A detailed comparison between the crystal morphologies of polymers and of monomeric hard spheres is currently in progress.

We have also described in detail two new descriptors of local structure, the characteristic crystallographic element norm and a geometric analysis based on the Voronoi cells. It is established that during crystallization the local environment around each site becomes more spherical and more symmetric. In turn, this shape transformation allows the sphere sites more freedom to move locally. Thus, the entropy of the system increases and it is the driving force for the crystallization of chain packings.

Present efforts include the modeling study of phase transition in athermal polymer packings under varied conditions of confinement, mainly through the presence of a hard wall. The proposed simulation approach is further generalized to treat polymer packings with a finite degree of chain stiffness.
